# Supplementary material for: 12.1 terabit/second data center interconnects using O-band coherent transmission with QD-MLL frequency combs
Source: Nat Commun. 2024 Sep 4;15:7741. doi: 10.1038/s41467-024-51682-2 (PMC11375058; doi:10.1038/s41467-024-51682-2)
Supplement: Supplementary file 1 — Supplementary Information [file 41467_2024_51682_MOESM1_ESM.pdf]

12.1 Terabit/Second Data Center Interconnects Using O-band Coherent Transmission with QD-MLL Frequency Combs – Supplement

Santiago Bernal[(1)*], Mario Dumont[(2)], Essam Berikaa[(1)], Charles St-Arnault[(1)], Yixiang Hu [(1)], Ramon Gutierrez Castrejon[(1,3)], Weijia Li[(1)], Zixian Wei[(1)], Benjamin Krueger[(4)], Fabio Pittalà[(4)], John Bowers[(2)], and David V. Plant[(1)]

[(1)] Dept. of Electrical and Computer Engineering, McGill University, Montreal QC H3A 0G4, Canada,
[(2)] Department of Electrical and Computer Engineering, University of California, Santa Barbara, CA 93106, USA,
[(3)] Inst. of Engineering, Univ. Nacional Autónoma de México UNAM, Cd. Uni., 04510 Mexico City, Mexico,
[(4)] Keysight Technologies Deutschland GmbH, Böblingen 71034, Germany
*Santiago.Bernal@mail.mcgill.ca

S1: Scaling DCI Capacities

Figure S1 details challenges and potential solution strategies for increasing DCI capacities to points defined on the Ethernet roadmap. Per Fig. S1, increasing capacity is achievable by i) increasing the symbol rate, and/or ii) increasing the modulation cardinality, and/or iii) space division multiplexing using more fibers. For IMDD, increasing the symbol rate is prohibitive near the edges of the O-band because of chromatic dispersion. Coherent transmission supports baud rate increases by leveraging advancements in both CMOS node shrinkage and S21 EO bandwidth increases in RF and electro-optic components. Because SNR requirements increase with increasing cardinality, commercially deployed solutions center around PAM4 and 16QAM for IMDD and coherent transmission, respectively.

A. Spatial division multiplexing (SDM): IMDD solutions are generally attractive for their lower cost, driven by the uncooled laser operation. To multiplex more channels without compromising the $\Delta\lambda$ of the CWDM grid and maintain the uncooled operation, multiplexing can be done by transmitting the same λs generated and loaded with data from independent transceivers over multiple fibers. This softens the chromatic dispersion limitations and preserves the required $\Delta\lambda$ for uncooled operation at the expense of extra fiber costs. The extra fiber can be a different SMF fiber that is commonly referred to as parallel single mode fiber (PSM), or it can employ the emerging multi-core fiber (MCF) technology. In both scenarios, the main limitation is the cost of the fibers, which scales with the length of the transmission link. It is unlikely that PSM will be a viable option beyond 2 km, because of the fiber costs. SDM is neutral to whether IMDD or coherent transceivers are deployed, it just scales the capacity by the number of parallel fibers.

B. Wavelength division multiplexing (WDM): In WDM, the different λs are multiplexed and transmitted over the same fiber by optical Mux/DeMux filters. Depending on the separation between the wavelengths, there are two WDM scenarios:

i) Coarse WDM; For $\Delta\lambda > 5$ nm, the CWDM grid is the standard for IMDD O-band transmission as it enables uncooled laser operation. With the large Δλ, it is assumed that the laser will not drift outside the allocated wavelength window for the operational temperature range inside the data center.

ii) Dense WDM; the wavelength spectrum is densely packed with signals at $\Delta\lambda \sim 1$ nm, and it is commonly used in coherent transmission deployment across the C and L wavelength bands. Given the narrow wavelength window per λ, using active laser tuning is mandatory to make sure that the laser does not drift outside the allocated slot. The optical Mux/DeMux filters used have a sharper roll-off factor and only tolerate laser wavelength shifts within ± 4 GHz.

This creates a 2x2 matrix, upon considering the possibility of employing IMDD or coherent transceivers. In the following sections, we discuss the four options in detail for scaling the capacity of intra-data center interconnects.

B.1) CWDM IMDD transmission: Given the chromatic dispersion limitations, let us assume that the capacity scaling is achieved via multiplexing more IMDD channels with smaller Δλ. The workable optical bandwidth of the O-band is ~ 40 nm; hence, multiplexing more than 8 channels will require shrinking the Δλ to less than 5 nm. This is extremely challenging unless all the lasers are cooled with independent TEC controllers to avoid drifting outside the narrowed Δλ. To reduce the number of required TECs, employing an optical comb source that generates more than 8 carriers with $\Delta\lambda \sim 2.5$ nm is advantageous as it requires a single TEC to align all the λs with their allocated slot. Yet, this optical frequency comb does not exist, and realizing a device with the outlined specifications is extremely challenging as discussed in Section 5 in the main text.

B.2) CWDM coherent transmission: Replacing the IMDD channels with coherent transmission with the same CWDM grid increases the capacity by 4 folds; hence, it can scale towards 3.2 and 6.4 Tbps. In addition, coherent transmission tolerates chromatic dispersion; hence, there is no fundamental limit on the symbol rate which can be further improved with the advancements in CMOS fabrication. The limitation in this solution is that coherent transmission requires active laser tuning with TECs to align the carrier and LO lasers, which considerably increases both cost and power consumption.

B.3) DWDM IMDD transmission: Employing DWDM in the O-band near the zero-dispersion wavelength alleviates the chromatic dispersion limitations, but it will require active laser tuning with multiple TEC controllers unless a frequency comb is used. In addition to this increase in cost and power, DWDM IMDD transmission will suffer from stronger fiber non-linearities as compared to the coherent case, in particular four-wave mixing (FWM). This can be ascribed to the absence of chromatic dispersion (See section of the main paper 4.2). Moreover, this option requires very sharp roll-off factor optical Mux/ DeMux filters because direct detection has no spectral reference.

B.4) DWDM coherent transmission: Active laser tuning is a requirement for coherent transmission, so it is not an extra overhead dictated by this architecture. In addition, employing an optical frequency comb, as reported in this work, can reduce the required number of TEC controllers to just 1 per n λs. The advantage of this pathway compared to the IMDD alternative is that coherent transmission suppresses the optical carrier; hence, it has more tolerance to fiber non-linearities as shown in Section of the main paper 4.2. In addition, coherent detection employs the LO as a spectral and phase reference; hence, the receiver can differentiate between the signal of interest and neighbor channel leakage. This enables employing cost-effective slow roll-off factor optical Mux/ DeMux filters and offers a scalable path for higher capacities.

Yet, the debatable question is whether to employ C-band or O-band in DWDM coherent transmission. Employing the C-band benefits from the already mature C-band coherent technologies, which reduces the development costs for the industry and enables the seamless migration of their technologies inside the data centers. In addition, the C-band introduces lower fiber non-linearities penalty to the performance because of the presence of chromatic dispersion. On the other hand, O-band operation benefits from the reduced DSP complexity by dispensing the DSP block dedicated to chromatic dispersion compensation. Therefore, technologically both options are feasible with minor differences. Since data center operators have preferred operating in the O-band for years, O-band could benefit from some momentum to build on.

C. Single-carrier coherent transmission: This path relies on the improvements in CMOS to push the capacity per λ to 1.6 Tbps and potentially 3.2 Tbps in the future. Yet, this path is not scalable beyond 3.2 Tbps (unless CWDM is employed). Increasing the symbol rate requires the most advanced CMOS node and RF bandwidth of more than 100 GHz, which increases the development costs. However, its main advantage is that it yields the least hardware count per Gbps.

Practically, scalability is achieved by combining multiple pathways/technologies. For instance, pure SDM will never be used without CWDM for cost reduction; hence, only 4 fibers are needed to achieve 3.2 Tbps with the current 4x200 Gbps CWDM4 grid. Yet, the same throughput can be realized with a single fiber employing coherent transmission over the same CWDM4 grid. According to this analysis, we think that DWDM coherent transmission with the development of power-efficient optical frequency combs offers a scalable path toward higher system capacities (i.e. 12.8 Tbps), with reduced power and cost overheads when compared with the alternative pathways. The research work presented in this contribution substantiates this statement.

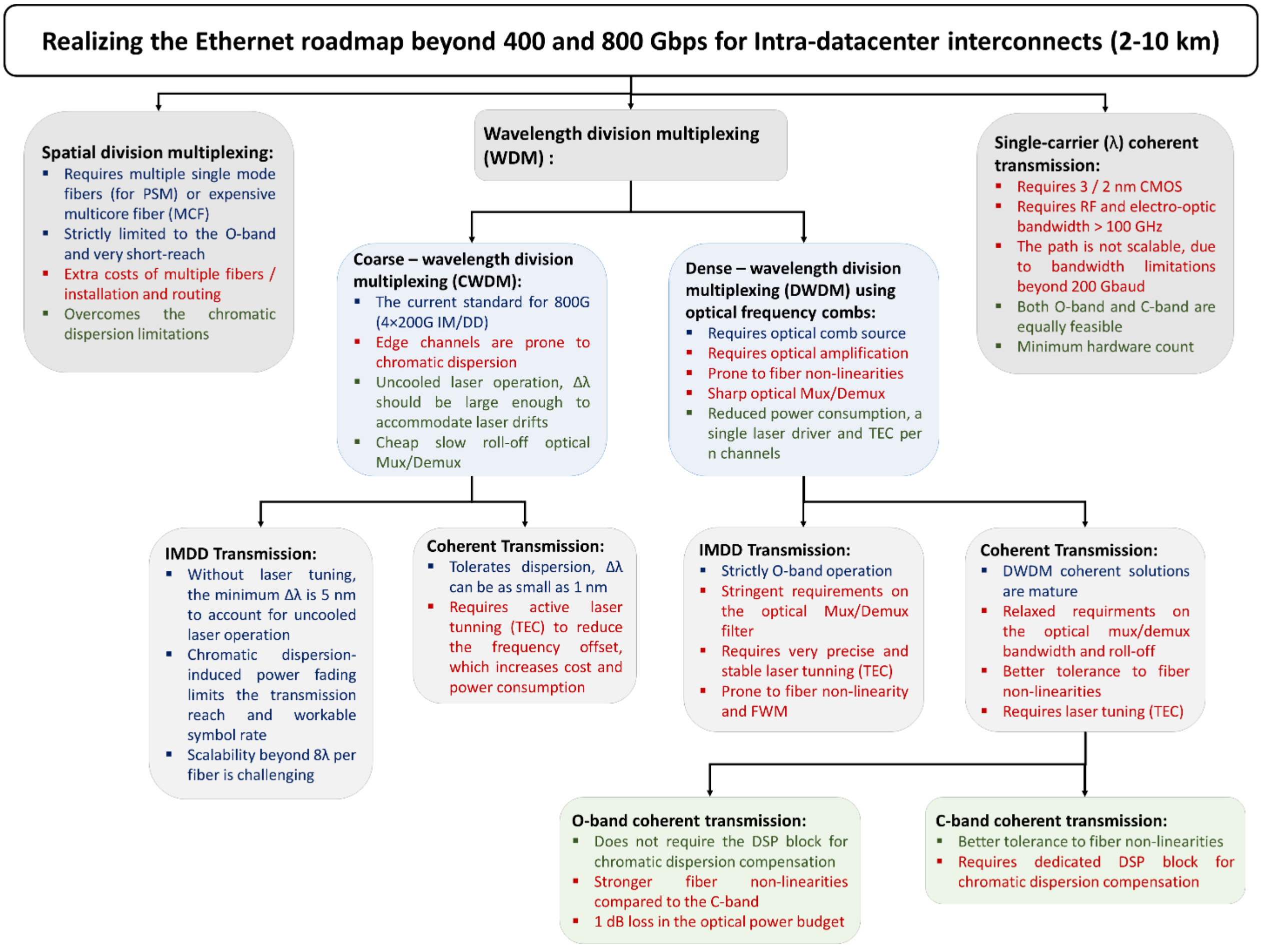

Fig. S1: Architectural analysis of the different transceiver architectures. Higher data rate transmission follow the Ethernet roadmap. The advantages (green) and disadvantages (red) of each solution are highlighted.

S2: QD-MLL Previous Coherent Transmission Demonstrations

Table S1 below shows the details of the literature survey carried out to support Fig. 1 of the main text. Our comparison was limited to coherent OFTS demonstrations using a Quantum-Dot (or dash) mode-locked laser [1-8].

Table S1: Summary of recent Comb-based coherent demonstrations.

**Reference**	**Number of lines**	**Baudrate [GBaud]**	**Modulation Format**	**Length of Fiber [km]**	**Optical band**	**Net rate per λ [Gbps]**	**Summation Net rate [Tbps]**	**Year**
[1]	38	38	16QAM	75	C-band	281	10.68	2019
[2]	60	20	32QAM	75	C-band	187	11.22	2020
[3]	47	32	16QAM	B2B	C-band	245	11.5	2019
[4]	56	28	16QAM	100	C-band	210	11.72	2022
[5]	48	28	16QAM	100	C-band	187	8.96	2020
[6]	23	45	QPSK	75	C-band	168	3.87	2019
[7]	179	40	16QAM	75	C-L-band	280	50.2	2017
[7]	94	40	16QAM	75	C-L-band	320	36.4	2017
[8]	160	23	64QAM	75	S-C-L-band	244	39.0	2020
This work	*26*	*56*	*16QAM*	*10*	*O-band*	*390*	*10.15*	*2024*
This work	*26*	*56*	*32QAM*	*10*	*O-band*	*467*	*12.14*	*2024*

S3: Comb-based DCIs using pluggables

Figure S2a illustrates the concept of a single QD-MLL enabling a coherent comb-to-comb optical transmission system using n-pluggables in a current data center rack configuration. This configuration is composed of a top rack with n-pluggables and a lower rack with the comb laser source. Fig. S2c shows the comb laser and demultiplexer which corresponds to a single rack of the concept shown in Fig. S2a. The system would only require one TEC to stabilize the laser source for all pluggables. The laser source output would be de-multiplexed and transmitted separately to all n-pluggables to be used as both carrier (outbound) and LO (inbound). This is shown in Fig S2a by the fibers connecting the bottom rack to the top rack. This would require an upgrade to the current pluggable form factors as the need for an external laser source would require a third optical interface, as compared with current QSFP standards. This form factor evolution is not uncommon as technologies improve as was seen with the introduction of QSFP replacing SFP form factors for higher Ethernet standards. As seen in Figure S1b, this 3-port pluggable would amplify the external laser source and split it to be used as a carrier and LO. A Tx PIC would modulate the signals and output them to be multiplexed by current WDM Tx filter racks to be propagated in a single fiber. The inbound signals would be de-multiplexed and transmitted to each receiver pluggable using the already existing WDM Rx filter racks. This concept would require careful planning of the layout of the different fibers in order to ensure a small form factor for DCI operations.

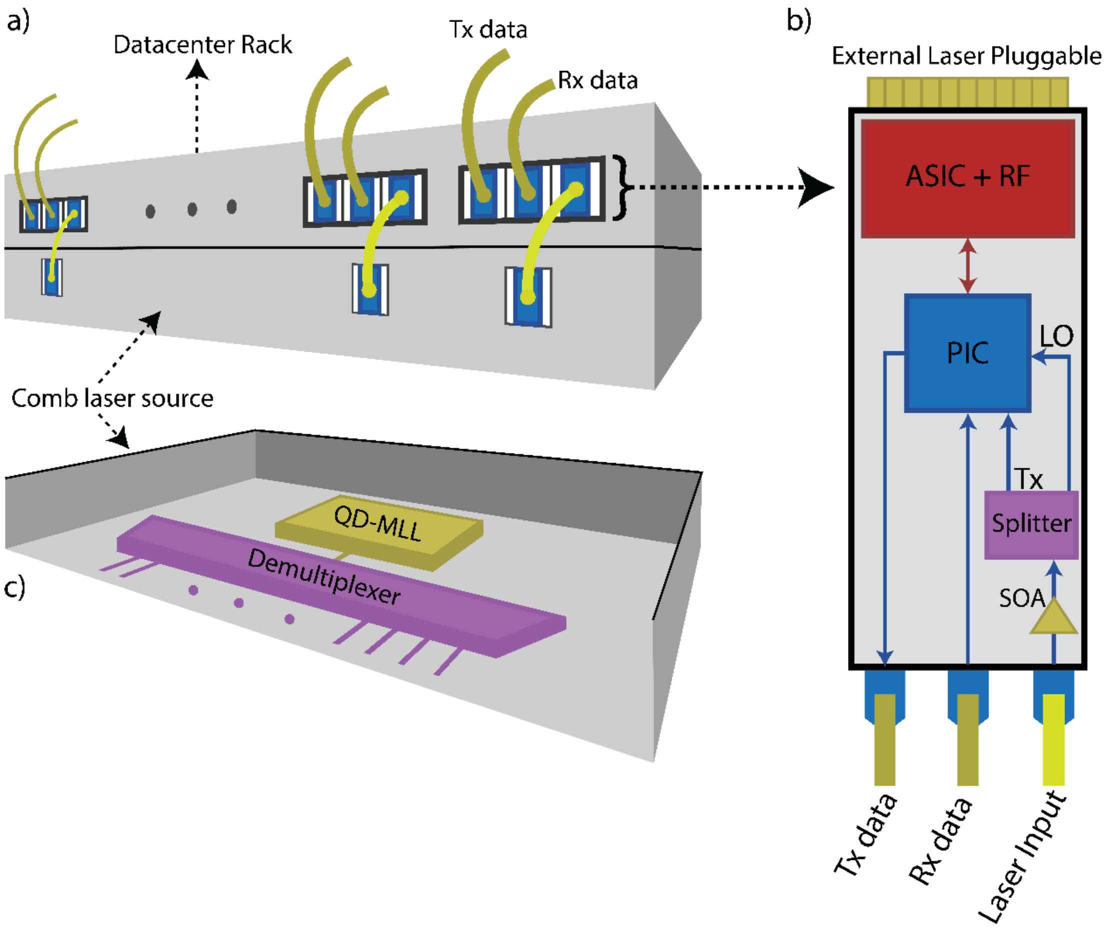

Fig. S2: Concept of a comb-to-comb coherent scheme consists of *n*-pluggables driven by one QD-MLL. The datacenter rack **a** is composed of *n* external laser pluggables **b** driven by a single QD-MLL **c**.

S4: O-band QD-MLL Fabrication and Characterization

A key enabler of this work is the development of O-band QD-MLL with high output optical power and sufficient optical bandwidth. This section describes the fabrication procedure and characteristics of the two O-band QD-MLLs used in this work as carrier and LO.

The lasers were grown by Molecular Beam Epitaxy (MBE) with a Varian GenII system on a 3-inch n-type 001 wafer. The indium arsenide (InAs) quantum dots (QDs) in the active region were grown by using the Stranski–Krastanov growth mode, which yields highly uniform QD nanostructures. Epitaxial structure is shown in Fig. S3(a); the active region contains six layers of QDs sandwiched in a 7 nm Indium Gallium Arsenide quantum wells, known as a Dots in a Well (DWELL) structure. Each Dwell layer is separated by 37.5 nm with Gallium Arsenide (GaAs)

spacers, which contain a p-modulation doped layer. P-doping in the active region is known to improve the gain and high-temperature performance of the laser[9]. The active region is placed between 50 nm GaAs waveguide layers. Above and below, there are 1.4 µm 40% Aluminum Gallium Arsenide (AlGaAs) cladding layers and highly doped GaAs contact layers, p-type and n-type respectively. This epitaxial stack is identical to that in ref. [10]. The lasers were fabricated by using standard dry etching and metallization processes [11] to form shallow-etched waveguides, identical to the structure in ref. [10], and are shown in Fig. S3(b). The laser ridges are etched to be 2.4 µm wide, because this is found to be the optimum in the tradeoff space between output power, coupling to fiber, and optical power per comb line. A 10 µm long section of the ridge is additionally etched 600 nm to remove the p-contact layer and part of the p-cladding, which electrically isolates part of the gain section. This section is reverse biased and acts as the saturable absorber (SA) of the mode locked laser (MLL). The SA is placed in the middle of the cavity to use the harmonic mode locking effect to double the comb line spacing from 29.15 GHz to 58.3 GHz. After the fabrication is completed, the substrate is thinned to 200 µm and the cavity is formed by cleaving.

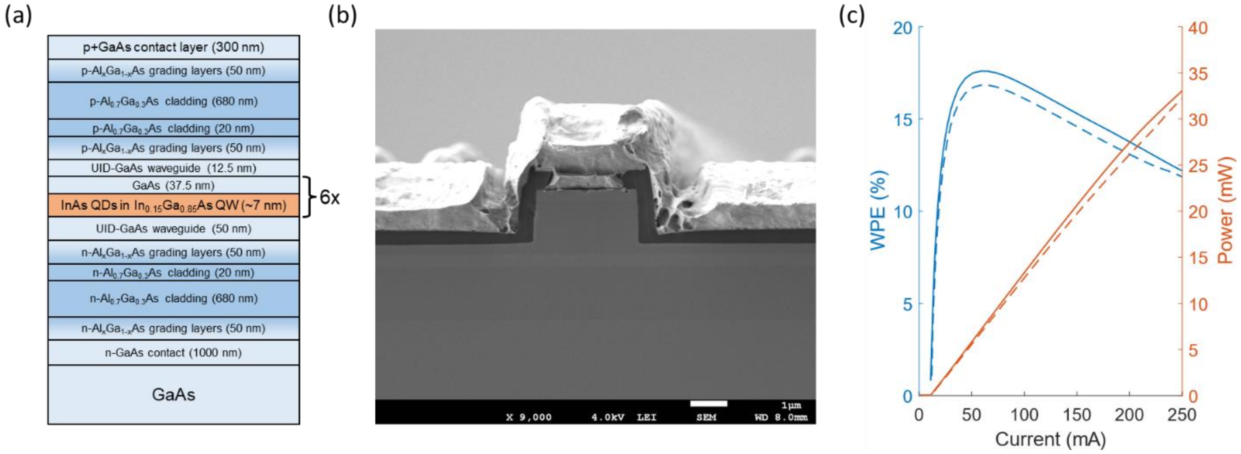

Fig. S3: Quantum-Dot Mode Locked Laser. a The epitaxial structure of the QD-MLL. **b** SEM of the fabricated QD-MLL. **c** The wall-plug efficiency and output power versus the bias current.

The performance of the devices is then measured to select known-good-devices. Light-current (LI) and wall plug efficiency performance of the two lasers used in this work are shown in Fig. S3(c) with the SA left floating. The devices are screened in the same manner as described in [12]. The injection current of the gain section and reverse bias voltage of the SA are stepped, and an optical spectrum and electrical spectrum are collected at each point. The fundamental 58.3 GHz beatnote of the comb is above 50 GHz, so an external local oscillator of 39 GHz and mixer are used to create

an intermediate frequency at 19.3 GHz. The RF beatnotes are fitted with a Voigt function to extract the linewidth, which is shown in Fig. S4(a) for both devices. The optical spectra are analyzed to establish the number of comb-lines in a 10 dB bandwidth, which is plotted in Fig. S4(b).

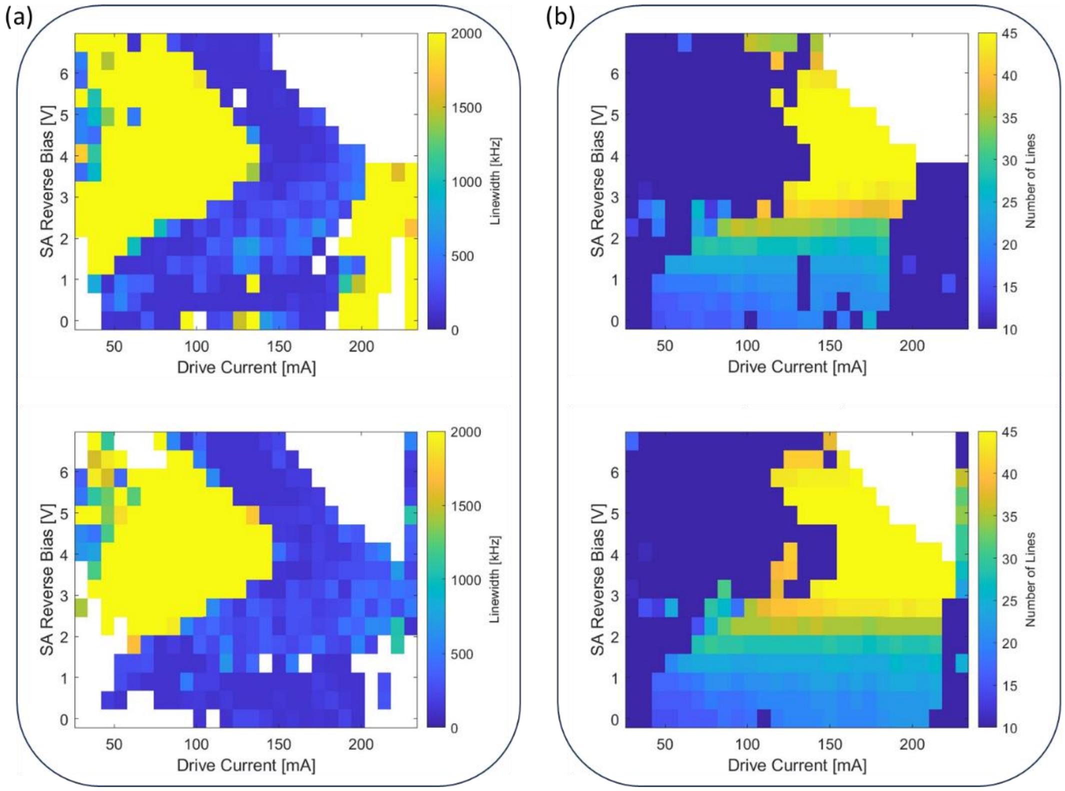

Fig. S4: QD-MLL characterisation for different comb states. a The linewidth characteristics and **b** number of comb lines in 10-dB bandwidth of the 2 QD-MLL employed in this work.

To find the optimum state for the data transmission experiment, each optical spectrum was analyzed to establish the 26 contiguous comb lines with the highest power. The power of the lowest line, highest line, and the difference between the lowest and highest power line (ΔP) are shown in Fig. S5 (a)-(c). These plots show similar information to the bandwidth in Fig.S4(b) but elucidates why the optimum lies at 2.4 V SA bias. Below this, the comb is too narrow, giving very low power in the edge states and very high power in the center. As the reverse bias increases, the bandwidth increases, and the ΔP approaches a minimum. However, above 2.4 V, the power of the 26th line and the 1st line both decreases, because the laser power is being spread over successively more comb lines while also decreasing the total output power. Therefor the optimum is achieved when the correct bandwidth is achieved to support 26 lines.

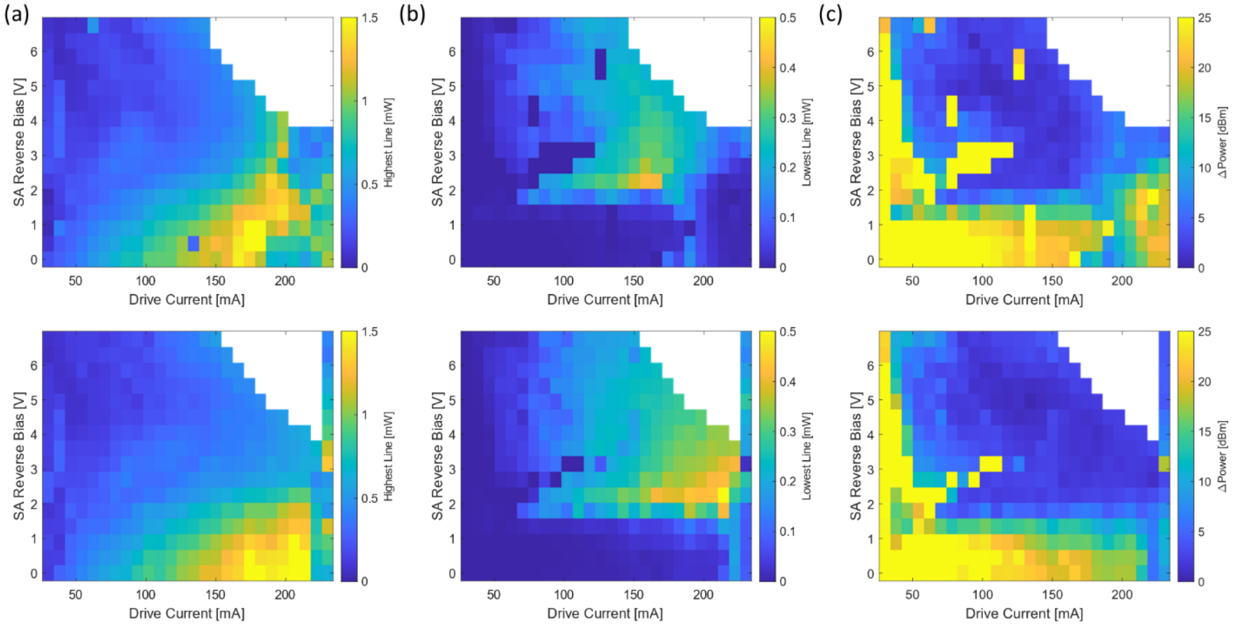

Fig. S5: QD-MLL power characterisation. The output power characteristics of the QD-MLLs used in the transmission experiment with the power of **a** highest line, **b** lowest line, and **c** difference in their power.

To analyze and project the performance of lasers of different lengths, the gain curve of one laser was measured in the same method described in [13]. The gain curve measured at the gain peak is shown in Fig. S6(a). Using the logarithmic gain model that is fit to the data[14], the threshold of lasers of different lengths can be approximated. The mirror loss was calculated using $\alpha_m = \frac{1}{L}\ln\left(\frac{1}{r_1 r_2}\right)$, where L is the cavity length and r_1 and r_2 are the E-field reflectivity values for each facet and are assumed to be 0.56. Using the measured internal loss, αi, accounting for the loss in the SA[15], the mirror loss is varied to find the laser threshold and threshold density as a function of the length as shown in Fig. S6(b). The Laser output is then calculated using the threshold and the slope efficiency given by $\frac{h\nu}{q}\eta_i\frac{\alpha_m}{\alpha_m+\alpha_i}$, and the current needed for 10 mW of output power (5 mW per facet) is shown in Fig. S6(c). This analysis does not consider effects from gain saturation or thermal degradation from self heating. This causes it to severely under-estimate the optimum cavity length for shorter laser cavities. However, it can be seen that the choice of length for our laser, 1375 µm operates close to the minimum of threshold, on the surplus gain side. It also approximates the current needed for 5 mW of output power per facet seen in the LI curve in Fig. S6(c). It is worth noting that the cavity length is inversely proportional to the comb lines spectral

separation; hence, our analysis indicates that the optimum comb spacing considering only the threshold current requirement is within 55 to 110 GHz. However, the gain saturation might be mor relevant for the smaller cavity lasers; yielding less output optical power than calculated.

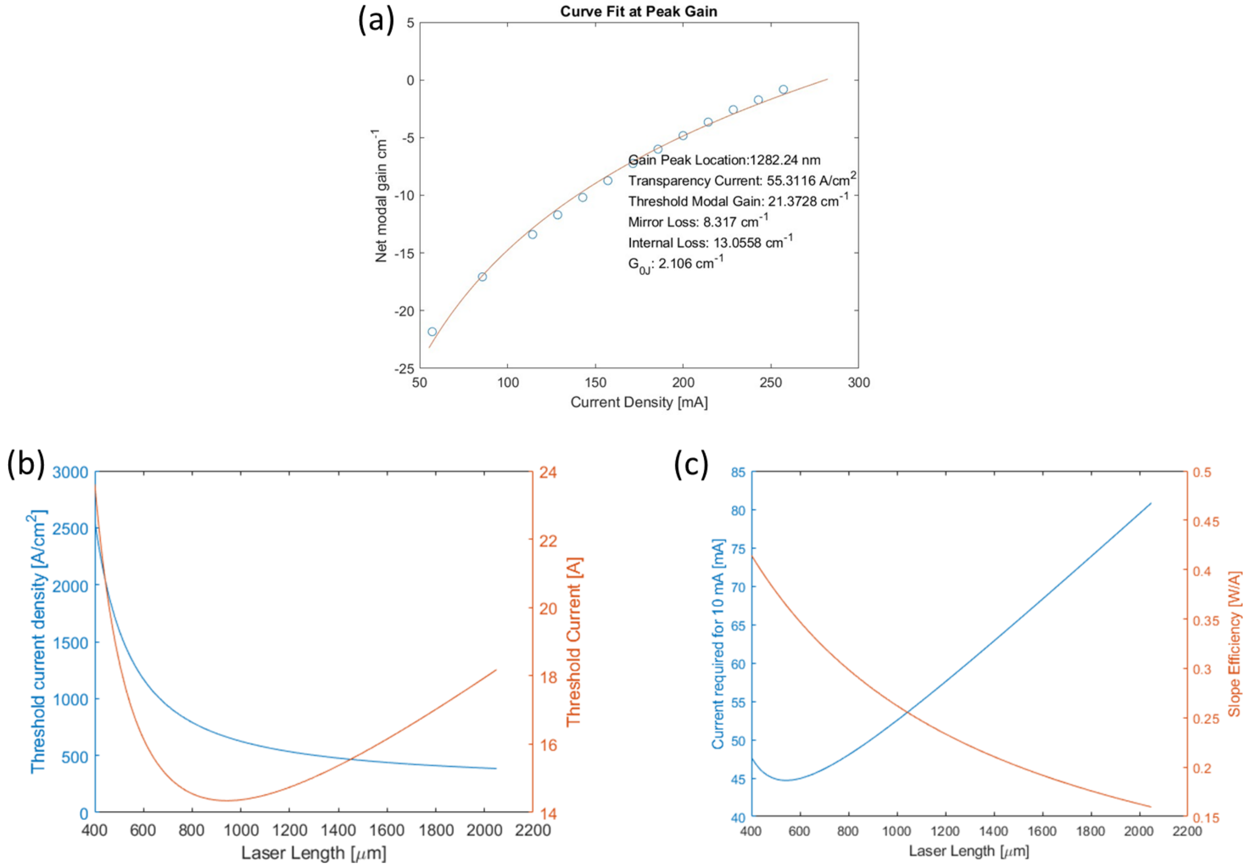

Fig. S6: QD-MLL characterisation for different cavity lengths. a The gain curve measured of one QD-MLL. **b** The calculated threshold current density and threshold current for a QD-MLL of the same structure that differs only in cavity length. **c** The current required and slope efficiency versus the cavity length assuming 10 mW (10 dBm) total output power per QD-MLL.

S5: Impact of Linear Cross-Talk on Comb Laser-Based IMDD and Coherent OFTSs.

We numerically investigated the nature and magnitude of the linear cross-talk penalty in the analyzed comb-based architectures using a similar simulation setup as described in the main text. Coherent and IMDD OFTSs were compared for 10 km reach and 56 GBaud transmission in the O-band and C-band. Only the SNR penalty of the central wavelength of the comb was analyzed since similar results are expected for other wavelengths. The linear XT was examined by varying different parameters of the optical Mux and DeMux pair while fixing the system channel spacing to 58 GHz.

The sensitivity to XT from neighbouring channels was numerically analysed by changing the DeMux bandwidth. The Mux and DeMux both used a brick-wall roll-off. The Mux bandwidth (BW) was set to 56 GHz while the DeMux BW was changed from 56 to 120 GHz, effectively allowing sweeping from 1 comb channel to 2 consisting of the entire central channel and half of each neighbouring channel. Fig. S7 shows the calculated SNR penalty for IMDD and coherent systems with varying DeMux BWs. Our results show the high sensitivity of IMDD to increasing DeMux BWs. The presence of neighbouring channels for BWs beyond 60 GHz interferes with the signal and causes a significant penalty. This is expected since in IMDD the direct detection process down-convert any signal that falls within the photodiode optical bandwidth to the same baseband; hence, any residual frequency component from a neighbouring channel will overlap with the signal of interest and significantly deteriorate the BER performance. In contrast, the coherent case is insensitive to the DeMux BW increase due to the presence of the LO [16]. In coherent detection, the LO laser acts as phase and frequency reference; thus, the down-converted signals will be spectrally referenced to the LO frequency and consequently the residual frequency components are rejected by the photodiode RF BW and digital filtering. Similar results were obtained in the C-band, making the choice between C and O-band immaterial for this analysis.

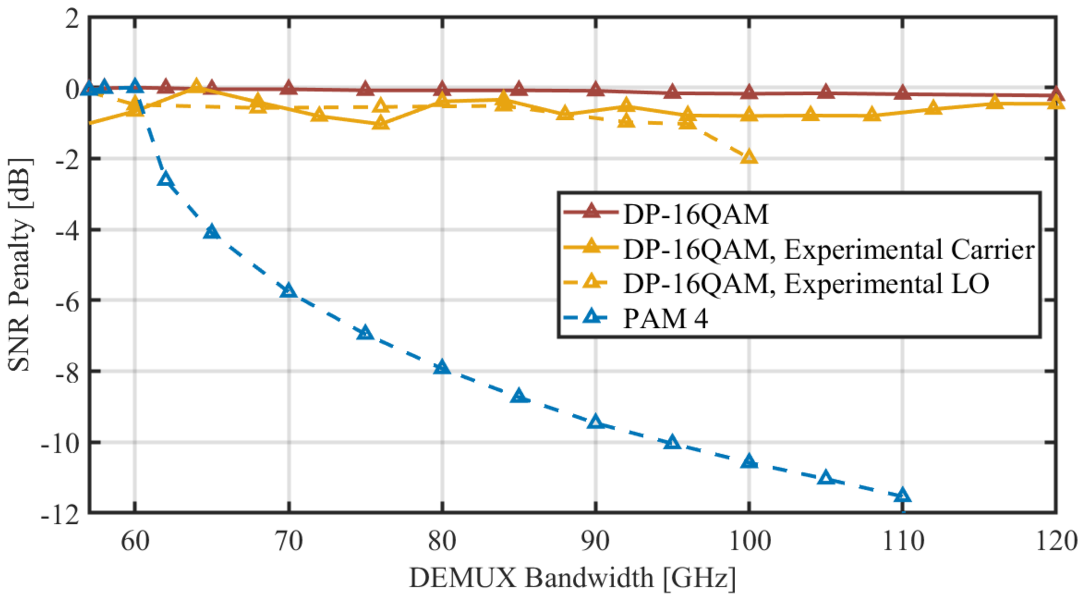

Fig. S7: Calculated SNR penalty as a function of the carrier and LO DeMux bandwidths. PAM4 and DP-16QAM modulation formats are shown at 56 GBaud after 10 km in the O-band. A comb source with 3 lines is used as the transmitter. A line spacing of 58 GHz and a DeMux brick-wall roll-off are used.

The DP-16QAM results were validated experimentally (solid yellow line) by changing the bandwidth of the EXFO TBF acting as DeMux at the receiver. The setup described in the main text was used with the addition of a second EXFO TBF before the optical hybrid. The increased 5 dB IL from this TBF decreased the throughput of the system. However, the results remain applicable to comb laser-based coherent systems using higher modulation formats such as DP-32QAM and DP-64QAM. The bandwidth of the Santec TBF is kept constant such that 3 comb lines are bulk modulated at 56 Gbaud, and the central line is optimized to mimic a DWDM scenario with 2 aggressor channels. The fiber length was kept as 10 km. The ROP at the balanced PDs was kept constant at +3 dBm using a constant LO power of +17 dBm. The same comb state as the main text was used for both lasers, operating with 160 mA driving current, 2.4V saturation absorber voltage, and 42 ºC. The temperature of the second comb was tuned to decrease the FO between the signal and LO comb lines under test. The FO was measured to be less than 250 MHz throughout the sweep. The bandwidth of the EXFO TBF filter at the receiver was changed from 56 to 124 GHz to mimic the sensitivity test of the de-multiplexer.

The sensitivity of the second comb laser acting as LO was also measured (dashed yellow line) by changing the bandwidth of the EXFO TBF at the LO. The same setup as above was used. The bandwidth of the Santec TBF is kept constant such that 3 comb lines are bulk modulated at 56 Gbaud, and the central line is optimized to mimic a DWDM scenario with 2 aggressor channels. The temperature of the second comb was tuned to decrease the FO between the signal and LO comb lines under test. The FO was measured to be less than 250 MHz throughout the sweep. The bandwidth of the EXFO TBF filter at the receiver LO is changed from 56 to 100 GHz to mimic a sensitivity test for the LO de-multiplexer. Fig. S7 shows the SNR penalty over the BW sweep. The SNR remains relatively constant as the LO TBF BW increases from 56 to 84 GHz. This is expected since the aggressor channels are being filtered completely. As the LO TBF BW continues to increase over 84 GHz, the neighboring channels start to affect the SNR of the system by saturating the amplifier and reducing the power per line of the LO comb line under test. Our results show that the sensitivity of the O-band LO DeMux is of ± 42 GHz in order to ensure operation below the FEC threshold.

The sensitivity of the comb-based system to temperature variations was also simulated and measured. This analysis is relevant because the temperature increase of the comb source results in an increase in the central wavelength of the laser. This will effectively red-shift all the comb lines. The simulations used a similar setup as described above and emulated the wavelength shift of the comb source due to temperature by simultaneously shifting the transmitter and Mux central wavelength. For the coherent case, the LO wavelength is kept constant, while for the PAM-4 case the DeMux central wavelength was kept constant. The Mux and DeMux BW were maintained at 56 GHz with a brick-wall roll-off. The central wavelength was shifted by 10 GHz to represent the effective shift in channel wavelength of the comb source given a 0.6 ºC temperature change. Fig. S8 shows the SNR penalty of the IMDD and coherent configurations as a function of the central wavelength shift which also represents the sensitivity of each system to potential comb source drifts. The penalty for IMDD increases almost linearly with the change in shifting. The coherent case is less sensitive to the frequency changes below 1 GHz, but quickly deteriorates as the shift increases. This can be explained by the fact that the useful information of the IMDD signal is duplicated in the two sidebands; hence, cutting a portion of one of the sidebands will reduce the SNR but no information is lost. On the contrary, coherent information is encoded in the double-

sideband signal; thus, cutting a portion of the signal corresponds to loss of information and significant performance deterioration.

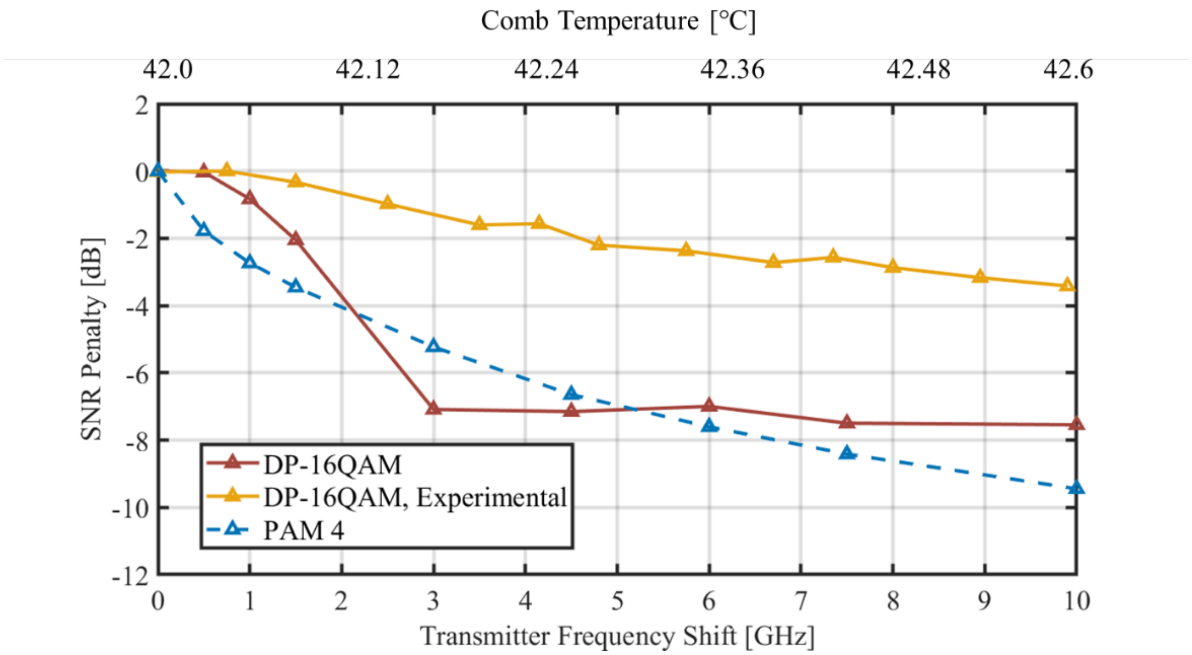

Fig. S8: Calculated SNR penalty as a function of a temperature-induced emission frequency shift. PAM4 and DP-16QAM modulation formats are shown at 56 GBaud after 10km in the O-band. A comb with 5 lines is used as a transmitter. A line spacing of 58 GHz is used in all cases.

These results were verified experimentally (yellow line) by using the same experimental setup as described above and changing the carrier comb source temperature to approximate large comb source drifts. The bandwidth and central wavelength of the receiver signal and LO filters are kept constant. The temperature of the LO comb laser and thus the emission wavelength is also kept constant. The penalty as the temperature increases is partially due to the increase in FO and the clipping of the signal due to the filter BW and tight channel spacing. The discrepancy between experimental and simulations results are due to the limitations of the experimental setup. More specifically, our setup bulk-modulates all channels, causing the neighboring channels to carry the same bit sequence as the channel under test, thus reducing the XT penalty in the experiment. Still, our results show that the sensitivity of the coherent system to temperature is smaller for small shifts of around ± 0.15 ºC as compared to IMDD. This shift is equivalent to a FO of around 800 MHz, which can be corrected through proper FO DSP.

The impact of the DeMux roll-off on the system performance was also investigated using the above-mentioned simulation infrastructure for coherent and IMDD cases. The corresponding SNR penalty is presented in Fig. S9. The Mux BW was set to 56 GHz, while the DeMux roll-off was changed from 0.0006 to 0.5. A root raised-cosine filter was employed. Our numerical results show a decrease of SNR as the roll-off factor grows, with a steeper slope for the IMDD case. The higher impact observed for PAM-4 modulation can be ascribed to the crosstalk from the neighbouring signals, which will overlap with the measured signal after photodetection. These results agree with those discussed in connection to Fig. S7, and their explanation follow similar lines. Equivalent results were obtained in the C-band, making the choice between C and O-band immaterial for this analysis.

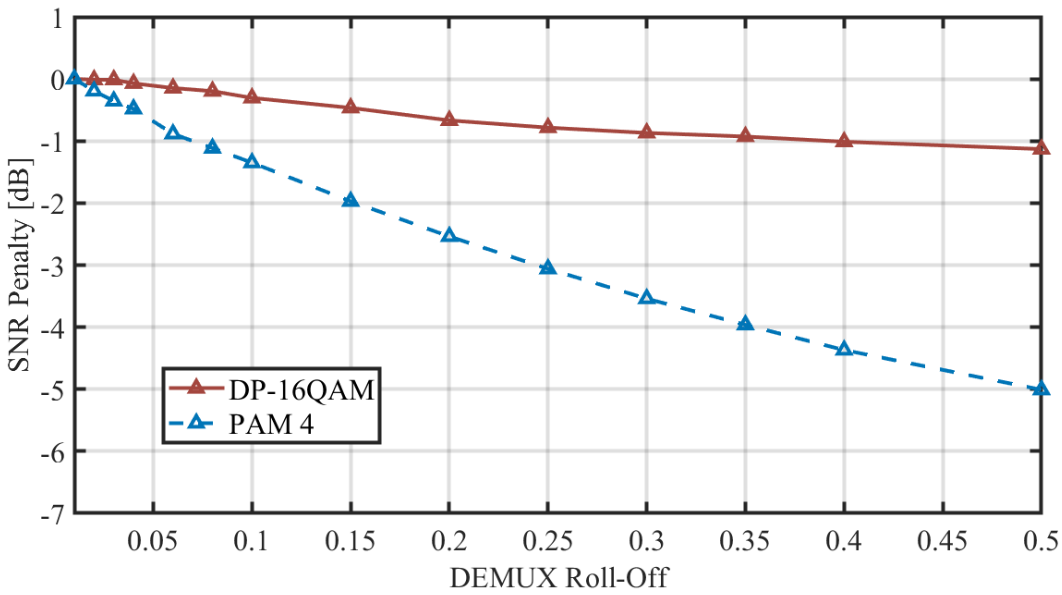

Fig. S9: Calculated SNR penalty as a function of the root raised-cosine DeMux roll-off factor. PAM4 and DP-16QAM modulation formats are shown at 56 GBaud after 10km in the O-band. A comb with 5 lines is used as a transmitter. A line spacing of 58 GHz is used in all cases.

Our numerical analysis was extended to investigate the XT penalty produced by varying the transmitted signal roll-off for coherent transmission. Fig S10 shows our results. In this case, the Mux and DeMux BW were kept constant with a BW of 56 GHz and a brick-wall roll-off. Our results show a small penalty for roll-off factors shorter than 0.1, but a steep decline as the tails of

neighboring channels start to leak into the channel under test. These results are almost equivalent for both the O and C-band cases.

These XT penalty results show the benefits of using coherent modulation for DWDM OFTS over IMDD as the tight channel spacings put restrictions on the bandwidth and roll-off of the Mux and DeMux components, the stabilisation of the comb source temperature, and the scale of the transmitter signal roll-off factor.

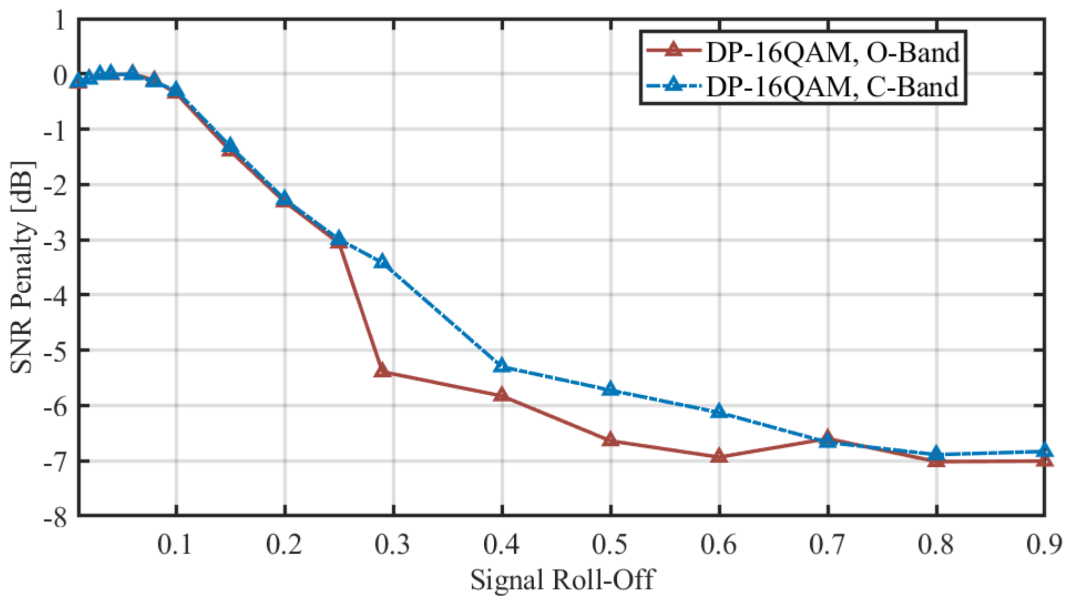

Fig. S10: Calculated SNR penalty as a function of the transmitter signal roll-off. DP-16QAM results are shown at 56 GBaud after 10km in the C and O-bands. A comb with 5 lines is used as a transmitter. A line spacing of 58 GHz is used in all cases.

S6: Impacts of Nonlinear Cross-Talk on Number of Comb channels for Coherent OFTS.

Figure S11 shows the calculated SNR penalty of adding multiple channels, each exhibiting the same average input power, to coherent OFTS (DP-16QAM). Our simulation used a similar setup as described above. The symbol rate was set to 56 GBd. The reach is 10km set in the O-band. Our results show the fiber non-linearity penalty incurred when multiplexing multiple high-power lines. Two values are considered: 0 and -10 dBm. The 4.5 dB penalty incurred when multiplexing 40 comb lines highlights one of the scaling issues with building a system with a high number of channels. The penalty for such a high number of channels can be mitigated by reducing the power per channel. However, such solutions are unlikely to have sufficient link budget for error-free operation.

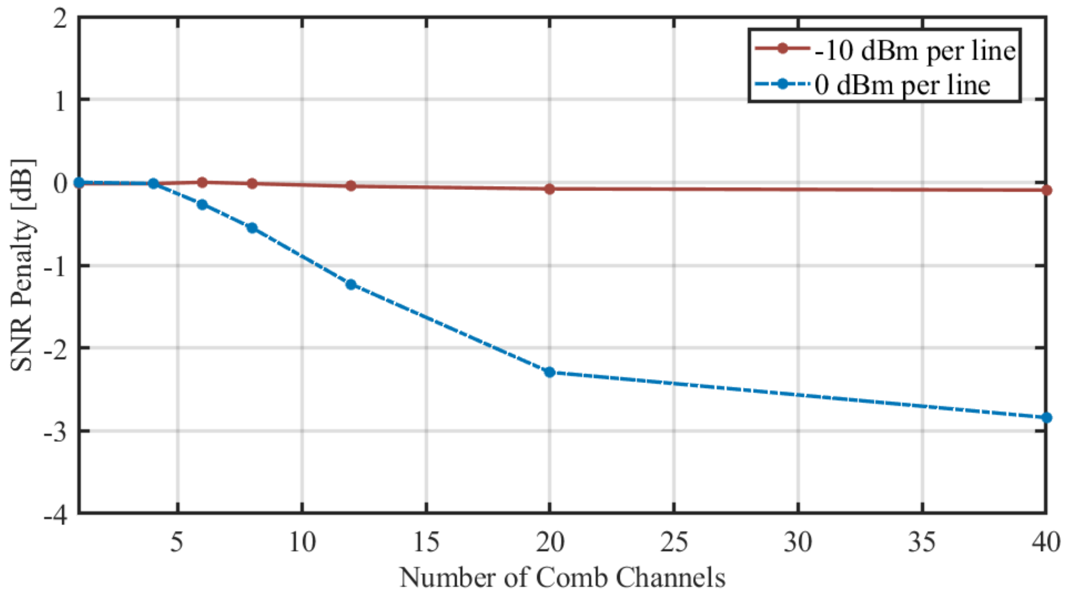

Fig. S11: SNR penalty calculation of fiber nonlinear effects. O-band coherent comb-based system is shown with varying number of comb channels present and power per line.

S7: FWM mitigation for comb-based IMDD OFTS.

Using orthogonal polarization launch of neighboring channels has been proven to reduce NL effects in DWDM systems[17]. Figure S12 shows the simulated improvement when using a XYYXXYYXXYYXXYYXXYYXXYYXX polarization configuration. Our results show a 3 dB improvement when using this method in our comb system with 26 lines over 10 km with a total optical power input into the fiber of 6 dBm. This method could be applied to an integrated solution by using on-chip polarisation rotators[18,19]. Alternatively, this solution can also be applied to a pluggable scheme (see Supplemental Section 3) by using external polarisation rotators as well as polarisation maintaining fibers before and after the mux and demux, respectively. While this penalty reduction is significant, it is not sufficient to surpass the coherent tolerance to FWM in comb-based OFTS. Additionally, our results show that PMD does not significantly impact the system performance in this use case.

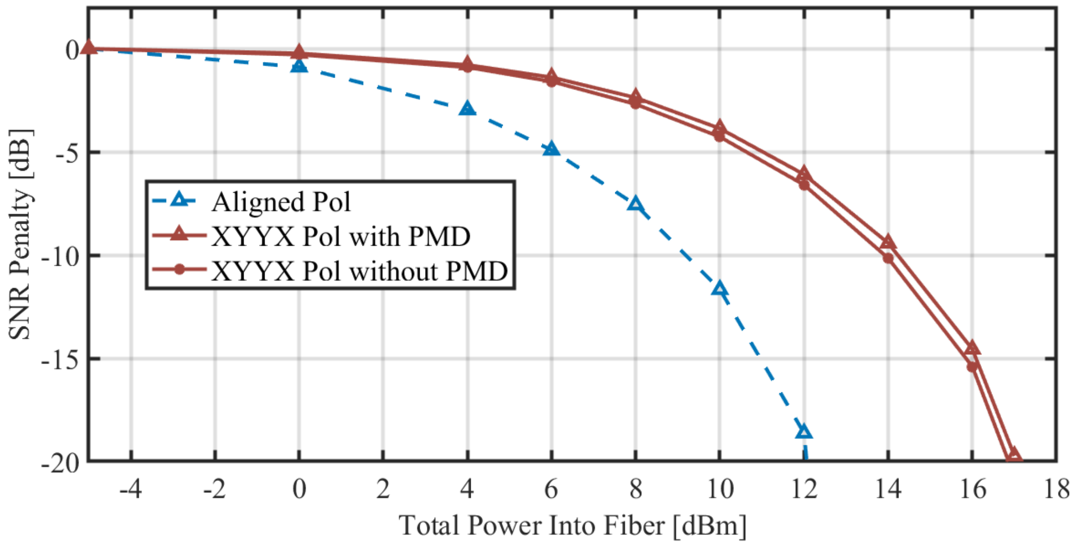

Fig. S12: SNR penalty calculation of fiber nonlinear effects for IMDD. O-band IMDD comb-based system is shown with varying polarisation configurations for neighboring channels as well as with and without the effects of PMD. A comb with 25 lines was used as transmitter with a line spacing of 58 GHz.

S8: Bulk modulation penalties for comb-based OFTS.

We performed simulations to calculate the difference between bulk and non-bulk modulated results for both IMDD and coherent. For IMDD, we found that the linear crosstalk was less than 0.1 dB due to the demux acting as a perfect bandpass filter. Figure S13 shows the calculated variations in SNR penalty as the central wavelength of the demux is shifted for a 56 Gbaud PAM 4 signal after 10 km. Our results show that the linear cross talk difference between bulk and non-bulk modulation in the IMDD case is only present when the neighbouring signals are also detected by the receiver. These results were experimentally validated as can be seen in Fig S14. In our IMDD experiment, we utilised a tunable bandpass filter (TBF) to act as a perfectly centered demux that mitigates the effects of neighboring channels at the receiver. Our results show that the BER improves when the neighboring signal present at the detector has the same bits as the device under test.

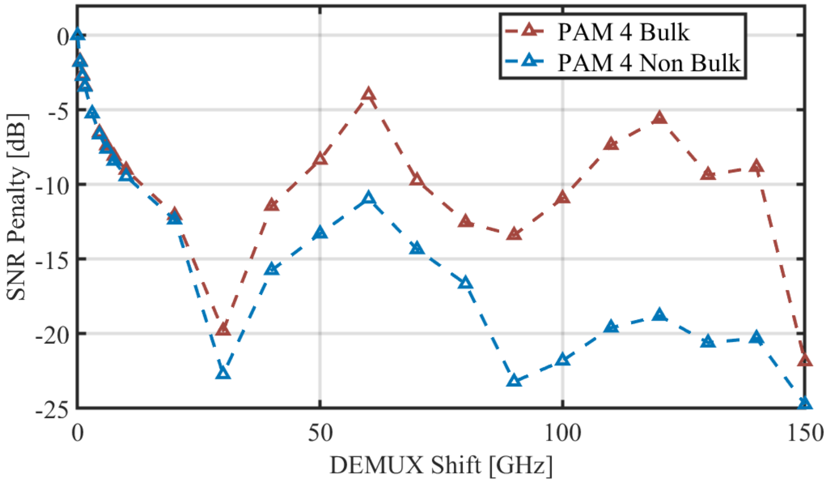

Fig. S13: Calculated BER penalty as a function of a demux frequency shift for either bulk or non-bulk modulated signals. A comb with 5 lines was used as transmitter with a line spacing of 58 GHz.

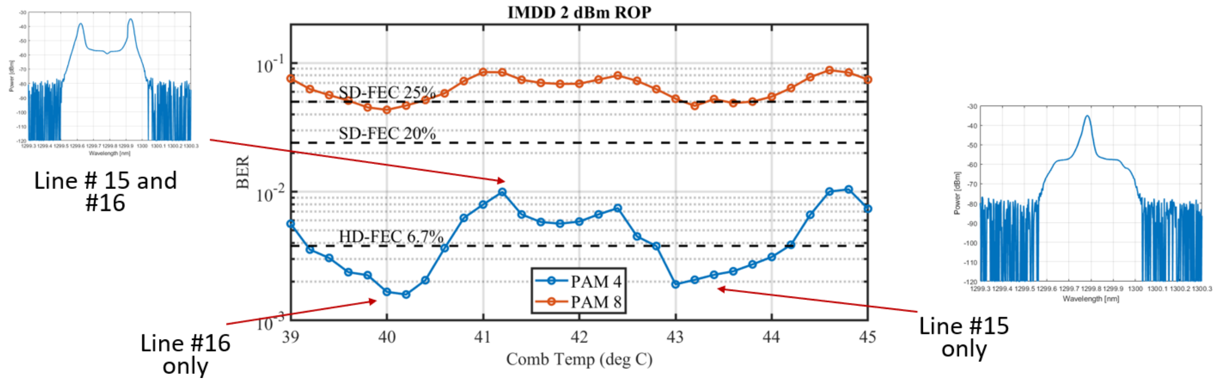

Fig. S14: Measured BER penalty as a function of a temperature-induced frequency shift for bulk modulated signals. A comb with 5 lines was used as transmitter with a line spacing of 58 GHz. Insets show the optical spectrum of received signal.

Our results also show that the linear cross talk is stronger for the bulk modulated signal in the coherent case. Figure S15 shows the calculated SNR penalty as a function of signal roll-off. As the roll-off increases, the SNR penalty increases due to the linear cross talk between neighbouring channels. For our experiment, we used a roll-off of 0.03 to minimize this effect which we calculated to be less than 0.05 dB. We would expect a slightly better performance if the experiment was done with a non-bulk modulated setup. Additionally, the presence of the LO acting as a bandpass filter enables the removal of any noise from the neighbouring channels as

can be seen in Fig S16. Our results show that the BER remains constant independently of the number of lines injected into the coherent receiver. This shows the tolerance of coherent detection to linear crosstalk. The variations in the BER are mainly due to measurement variations for that experiment.

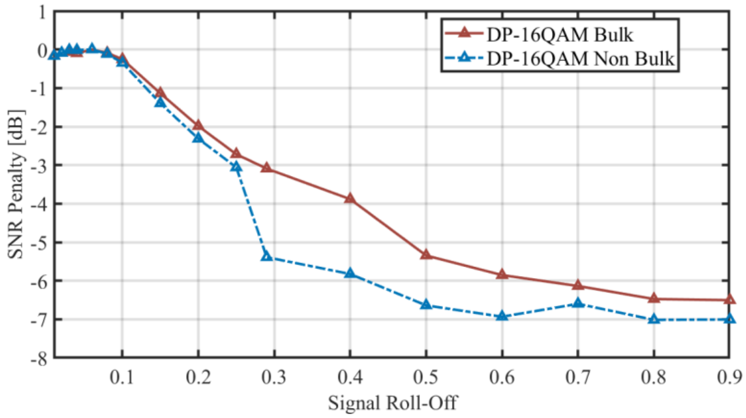

Fig. S15: Calculated BER penalty as a function of a Tx roll-off for either bulk or non-bulk modulated signals. A comb with 5 lines was used as transmitter with a line spacing of 58 GHz.

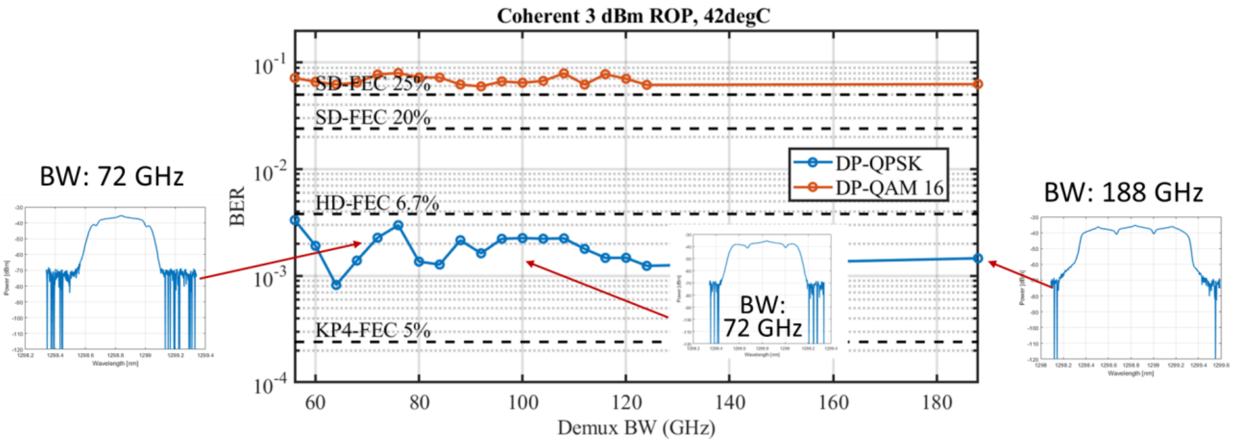

Fig. S16: Measured BER penalty as a function of a demux bandwidth increase for bulk modulated signals. A comb with 5 lines was used as transmitter with a line spacing of 58 GHz. Insets show the optical spectrum of received signal.

S9: Experimental setups used

Supplemental Figure S17 shows the coherent experimental system that is a modified version of the system used in [20,21]. Figure S18 shows the IMDD optical fiber transmission system (OFTS) experimental setup.

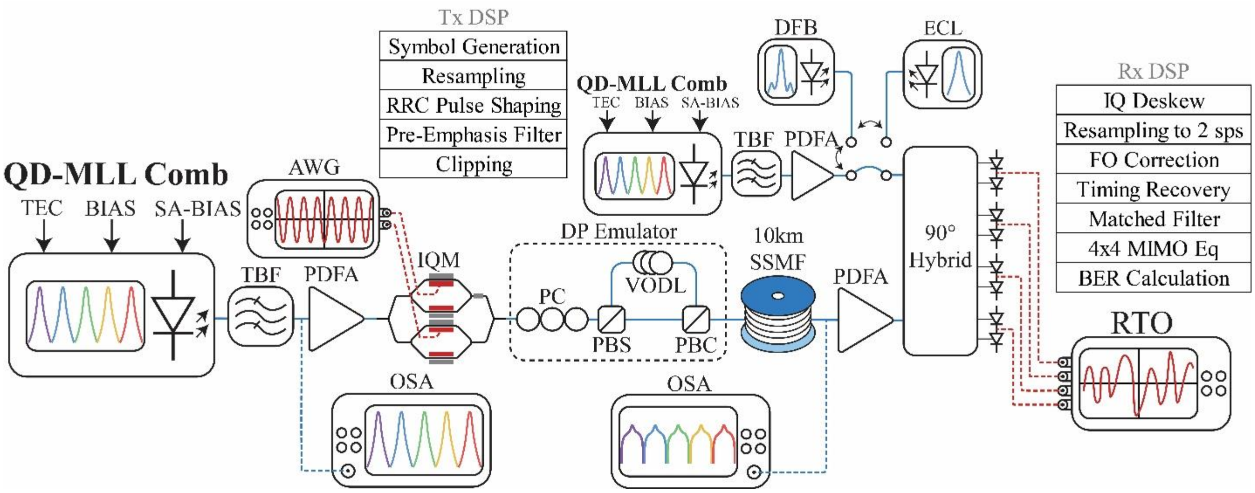

Fig. S17: Coherent experimental setup. The schematics shows the setup used for the transmitter (Tx) and receiver (Rx) and the DSP stacks used for each. The LO used varied from an ECL, a series of DFBs, or a second comb laser.

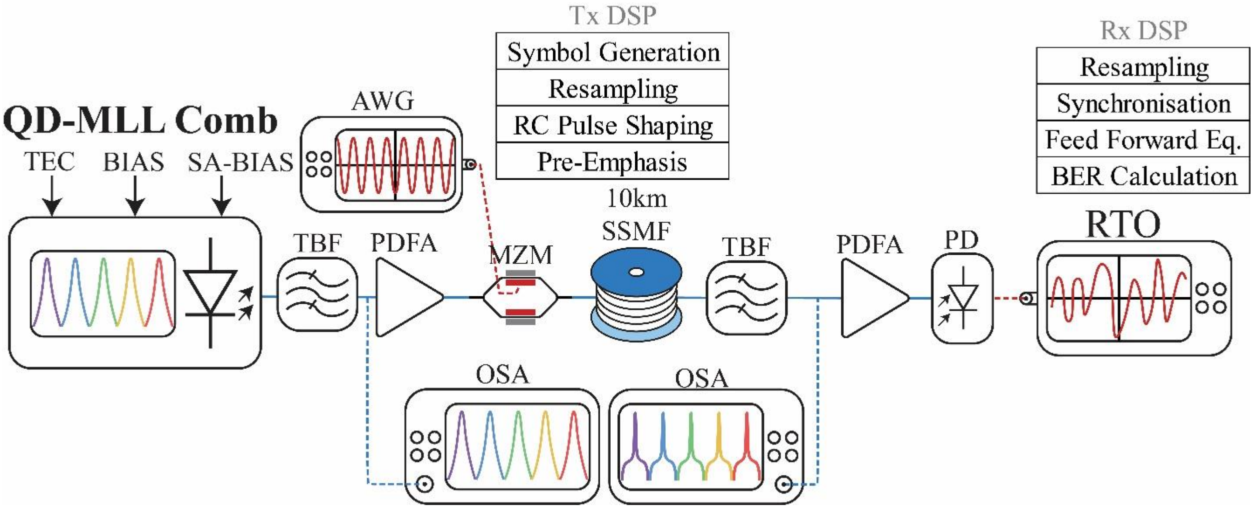

Fig. S18: IMDD experimental setup. The schematic shows the setup used for the transmitter (Tx) and receiver (Rx) and DSP stacks used.

Table S2: Summary of Net Bitrate Achieved for 10 km, O-band IMDD and coherent OFTS.

Modulation format	Baudrate [Gbaud]	Number of channels (under FEC)	FEC Overhead	FEC	Net bit rate per λ [Gbps]	Summation Net bit rate [Tbps]
PAM 4	56	26	6.7 %	HD-FEC	105	2.73
PAM 8	56	26	25 %	SD-FEC	134	3.49
DP-16QAM	56	26	14.8 %	O-FEC	390	10.18
DP-32QAM	56	26	20 %	SD-FEC	467	12.14

References:

1 Marin-Palomo, P. *et al.* Comb-based WDM transmission at 10 Tbit/s using a DC-driven quantum-dash mode-locked laser diode. *Opt. Express* **27**, 31110-31129 (2019). https://doi.org/10.1364/OE.27.031110

2 Kemal, J. N. *et al.* 32QAM WDM transmission at 12 Tbit/s using a quantum-dash mode-locked laser diode (QD-MLLD) with external-cavity feedback. *Opt. Express* **28**, 23594-23608 (2020). https://doi.org/10.1364/OE.392007

3 Lu, Z. *et al. 12.032 Tbit/s coherent transmission using an ultra-narrow linewidth qantum dot 34.46-GHz C-Band coherent comb laser*. Vol. 10947 PWO (SPIE, 2019).

4 Mao, Y. *et al.* Ultralow Noise and Timing Jitter Semiconductor Quantum-Dot Passively Mode-Locked Laser for Terabit/s Optical Networks. *Photonics* **9**, 695 (2022).

5 Liu, G. *et al.* Mode-Locking and Noise Characteristics of InAs/InP Quantum Dash/Dot Lasers. *Journal of Lightwave Technology* **41**, 4262-4270 (2023). https://doi.org/10.1109/JLT.2023.3244777

6 Kemal, J. N. *et al.* Coherent WDM transmission using quantum-dash mode-locked laser diodes as multi-wavelength source and local oscillator. *Opt. Express* **27**, 31164-31175 (2019). https://doi.org/10.1364/OE.27.031164

7 Marin-Palomo, P. *et al.* Microresonator-based solitons for massively parallel coherent optical communications. *Nature* **546**, 274-279 (2017). https://doi.org/10.1038/nature22387

8 Corcoran, B. *et al.* Ultra-dense optical data transmission over standard fibre with a single chip source. *Nature Communications* **11**, 2568 (2020). https://doi.org/10.1038/s41467-020-16265-x

9 Zhang, Z. *et al.* Effects of modulation p doping in InAs quantum dot lasers on silicon. *Applied Physics Letters* **113** (2018).

10 Dong, B. *et al.* Broadband quantum-dot frequency-modulated comb laser. *Light: Science & Applications* **12**, 182 (2023).

11 Wan, Y. *et al.* in *CLEO: Science and Innovations.* SW3Q. 3 (Optica Publishing Group).

12 Dumont, M., Liu, S., Kennedy, M. & Bowers, J. High-efficiency quantum dot lasers as comb sources for DWDM applications. *Applied Sciences* **12**, 1836 (2022).

13 Jung, D. *et al.* Highly reliable low-threshold InAs quantum dot lasers on on-axis (001) Si with 87% injection efficiency. *ACS photonics* **5**, 1094-1100 (2017).

14 Coldren, L. A., Corzine, S. W. & Mashanovitch, M. L. *Diode lasers and photonic integrated circuits*. (John Wiley & Sons, 2012).

15 Dong, B. *et al.* 1.3-µm passively mode-locked quantum dot lasers epitaxially grown on silicon: gain properties and optical feedback stabilization. *Journal of Physics: Photonics* **2**, 045006 (2020).

16 Savory, S. J. Digital coherent optical receivers: Algorithms and subsystems. *IEEE Journal of selected topics in quantum electronics* **16**, 1164-1179 (2010).

17 Liu, X. & Fan, Q. Inter-Channel FWM Mitigation Techniques for 800G-LR4, 1.6T-LR8, 400G-ER4 and 5G Fronthaul Applications Based on O-band WDM. *Journal of Lightwave Technology* **PP**, 1-10 (2023). https://doi.org/10.1109/JLT.2023.3316008

18 Sun, B. *et al.* On-chip beam rotators, adiabatic mode converters, and waveplates through low-loss waveguides with variable cross-sections. *Light: Science & Applications* **11**, 214 (2022). https://doi.org/10.1038/s41377-022-00907-4

19 Guan, H., Fang, Q., Lo, G. Q. & Bergman, K. High-Efficiency Biwavelength Polarization Splitter-Rotator on the SOI Platform. *IEEE Photonics Technology Letters* **27**, 518-521 (2015). https://doi.org/10.1109/LPT.2014.2384451

20 Berikaa, E. *et al.* Next-Generation O-band Coherent Transmission for 1.6 Tbps 10 km Intra-Datacenter Interconnects. *Journal of Lightwave Technology* (2023).

21 Berikaa, E. *et al.* in *Optical Fiber Communication Conference.* Th4B. 1 (Optica Publishing Group).
